# Sleeping Medications and Prevention of Functional Disability in Older Adults with Low Care Needs: A Prospective Cohort Study

**DOI:** 10.31662/jmaj.2025-0534

**Published:** 2026-03-27

**Authors:** Naoko Otsuki, Ryohei Yamamoto, Ayumi Kono

**Affiliations:** 1 Department of Community-based Integrated Care Science, School of Nursing, Osaka Metropolitan University, Osaka, Japan; 2 Health and Counseling Center, The University of Osaka, Toyonaka, Japan

**Keywords:** older adults with low care needs, functional disabilities, long-term care, claims data

## Abstract

**Introduction:**

Sleeping disorders, a common health problem among older adults worldwide, are a risk factor for functional disability. Although sleeping medications are extensively used to treat sleep disorders in older adults, their safety and efficacy in terms of cognitive and physical effects are controversial. The present 3-year prospective cohort study clarifies the association between sleeping medication and the incidence of functional disability in 1,652 older adults aged ≥65 years with low care needs in Japan’s long-term care insurance system (LTCI).

**Methods:**

We conducted a prospective cohort study using data from the Southern Osaka Health and Aging Study. The present study included 1,291 participants aged 65 years at the baseline date after excluding 302 (20.0%) participants who received long-term care certification during the six-month baseline period. The exposure was sleeping medication use. The outcome was the identification of functional disabilities based on LTCI certification. The association between sleeping medication use and the incidence of functional disabilities was assessed using unadjusted and adjusted Fine and Gray proportional subdistribution hazards models.

**Results:**

During the median observational period of 2.5 years, the incidence of functional disabilities was observed in 504 (54.6%) and 184 (49.7%) participants in the “No use” group and the “Use” group, respectively. This study clarified that the group using sleeping medications had a significantly lower risk of functional disability than the group not using them (sub-hazard ratios of “No use” and “Use”: 1.00 [reference], 0.82 [confidence intervals, 0.68-0.99]).

**Conclusions:**

Our findings differ from those of previous studies and may provide useful suggestions for home care services for older adults with low care needs. Managing sleep with professional support, such as assessing sleeping medication doses, may help prevent functional disabilities in individuals with low care needs.

## Introduction

The rapid aging of the population worldwide has led to an increase in older adults with complex conditions and a consequent rise in older adults with high care needs ^(1)^. Functional disabilities requiring care are major indicators of health in older adults and are risk factors for a wide range of adverse health outcomes, including hip fracture ^(2)^, frailty ^(3)^, obesity ^(4)^, cardiovascular disease ^(5)^, end-stage kidney disease ^(6)^, dementia ^(7)^, and even mortality ^(8), (9)^. In addition, functional disabilities lead to a decline in quality of life in older adults ^(10)^. Although preventive care for older adults is actively implemented in Japan ^(11)^, older adults with low care needs, such as those with frailty, remain a high-risk population for progression to functional disabilities ^(12)^. Therefore, preventing functional disabilities in this population is important for maintaining health and well-being.

Sleeping disorders are common among older adults worldwide ^(13), (14), (15)^ and are recognized risk factors for functional disabilities ^(16)^. Both sleep disorders and the use of sleeping medications have been associated with falls and hip fractures in nursing home residents, suggesting that sleep management may influence functional outcomes ^(17)^. Although sleeping medications are widely used in older adults ^(15)^, their physical safety remains controversial ^(17), (18)^. A study of 8,044 older adults (mean age: 82 years), who were able to walk but of whom 30.0% required assistance, reported no major adverse effects of melatonin receptor agonists ^(19)^, whereas another study of 5,009 adults aged ≥75 years, 63.6% of whom had care needs such as Parkinson’s disease, found an association between melatonin receptor agonist use and hip fracture incidence ^(20)^. These inconsistent findings suggest that the safety of sleeping medications may depend on background factors, including functional status. Older adults with low care needs are at high risk for functional disabilities and falls, and over 60% reportedly have sleeping disorders ^(18)^. Therefore, examining the association between sleeping medication use and functional disabilities in this population is warranted.

The present 3-year prospective cohort study clarified the association between the use of sleeping medications and the incidence of functional disability in 1,652 older adults aged ≥65 years with low care needs in Japan’s long-term care insurance (LTCI) system. The findings of the present study may offer clinically useful evidence for establishing effective strategies to prevent functional disability in high-risk populations.

## Materials and Methods

### Study design and data source

**Figure 1.**
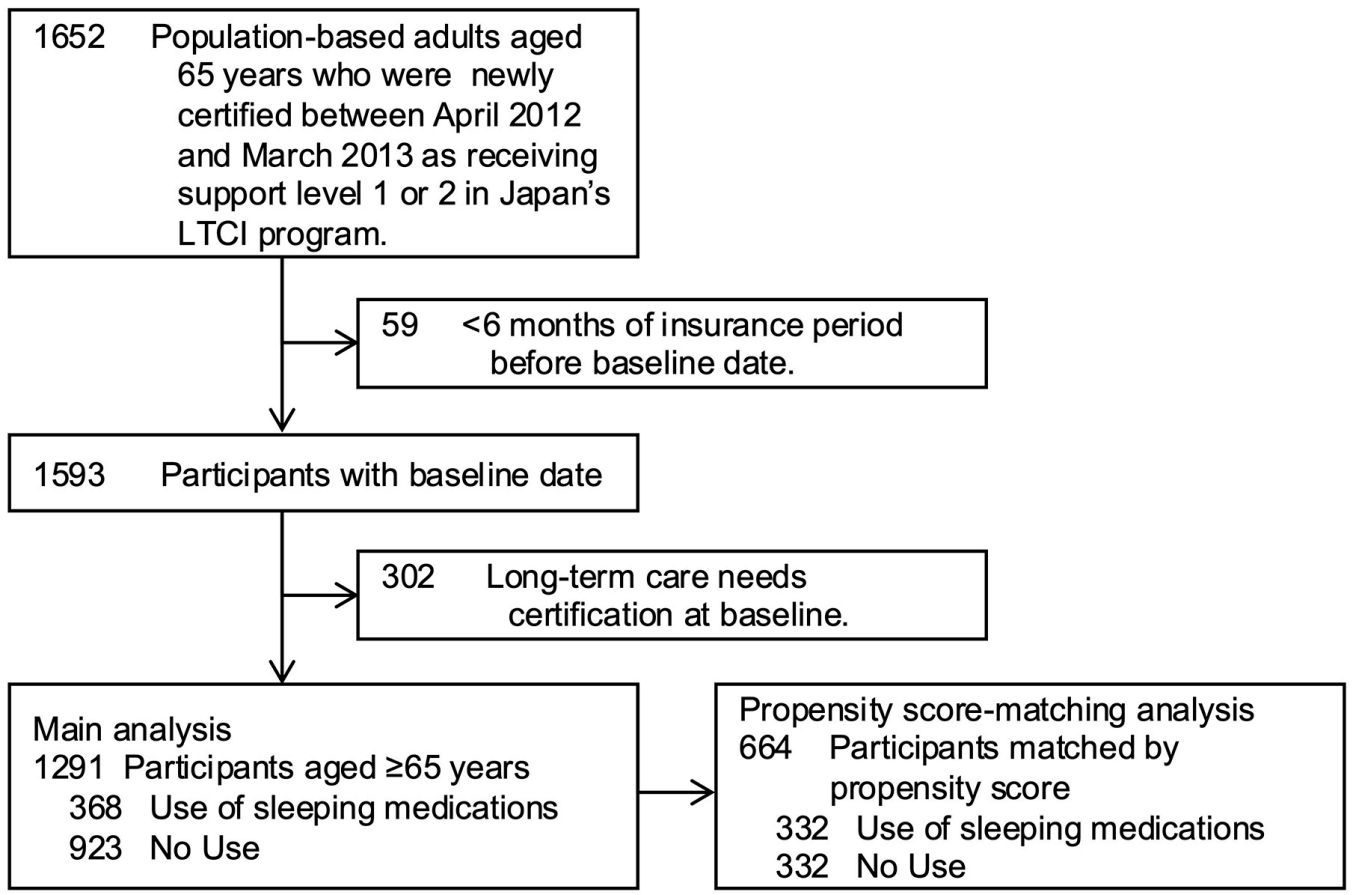
A flow diagram of this study entry.

We conducted a prospective cohort study using data from the Southern Osaka Health and Aging (SOHA) Study ^(12)^. Eligible participants in the SOHA study included population-based adults aged ≥65 years who were newly certified between April 2012 and March 2013 as requiring support level 1 or 2 in Japan’s LTCI system. In Japan, the Care Needs Certification Board of the municipal government certified long-term care needs and assigns care levels into seven categories: support levels 1 and 2 and long-term care 1, 2, 3, 4, and 5 ^(21), (22)^. The study setting comprised three municipalities (Izumi, Izumiotsu, and Misaki) in Osaka Prefecture, Japan. The average proportion of adults aged ≥65 years in these municipalities was 24.3%, similar to the national average of 24.1%, as of March 2013 ^(12)^. A total of 1,652 adults aged ≥65 years were newly certified between September 2012 and March 2013 as requiring support level of 1 or 2 in the LTCI program. Eligible participants were defined as older adults with low care needs. The baseline date was set as six months after the certification month for each individual, as baseline variables were measured using medical and long-term care insurance claims collected during those six months. The present study enrolled 1,593 participants at baseline after excluding 59 participants (3.6%) who died or lost insurance eligibility. Finally, 1,291 participants aged ≥65 years at baseline were included after excluding 302 (20.0%) participants who received long-term care certification during the six-month baseline period (Figure 1). As this study used medical claims data, none of the participants had missing baseline variables.

All insurance, medical, and LTCI claim data were retrieved from the National Health Insurance Database of Japan (Kokuho database: KDB).

### Measurements

The exposure in this study was determined based on the history of one or more prescriptions for sleeping medications during the six months preceding the baseline date. Baseline variables included age, sex, care levels, drug use, kidney replacement therapy, and region (Izumi City, Izumiotsu City, and Misaki Town). The baseline care level was determined by the certified support level in the LTCI at study entry. Drug use was determined based on a history of one or more prescriptions during the six months before the baseline. Medical claims codes for drugs were converted to Anatomical Therapeutic Chemical (ATC) Classification System codes using master data developed by the Japan Pharmaceutical Information Center (Shibuya-Ku, Tokyo, Japan) to identify sleeping medications (ATC codes: N05C), antianxiety drugs (N05BA, N05BB, and Kampo Yokukansan) ^(23)^, antidepressant drugs (N06AA, N06AB, N06AX), acetaminophen (N02BE), antidiabetic drugs (A10) ^(24)^, antiplatelet drugs excluding heparin (B01AC) ^(25)^, beta-blockers (C07) ^(24)^, calcium channel blockers (C08) ^(24)^, renin-angiotensin system blockers (C09) ^(24)^, cholesterol-lowering drugs (C10AA, C10AX09, and C10BA) ^(26)^, and antidementia drugs (N06DA and N06DX01) ^(6)^. The requirement for kidney replacement therapy, such as hemodialysis, peritoneal dialysis, or kidney transplantation was determined based on the history of one or more medical claims within six months before the baseline date ^(6)^.

**Figure 2.**
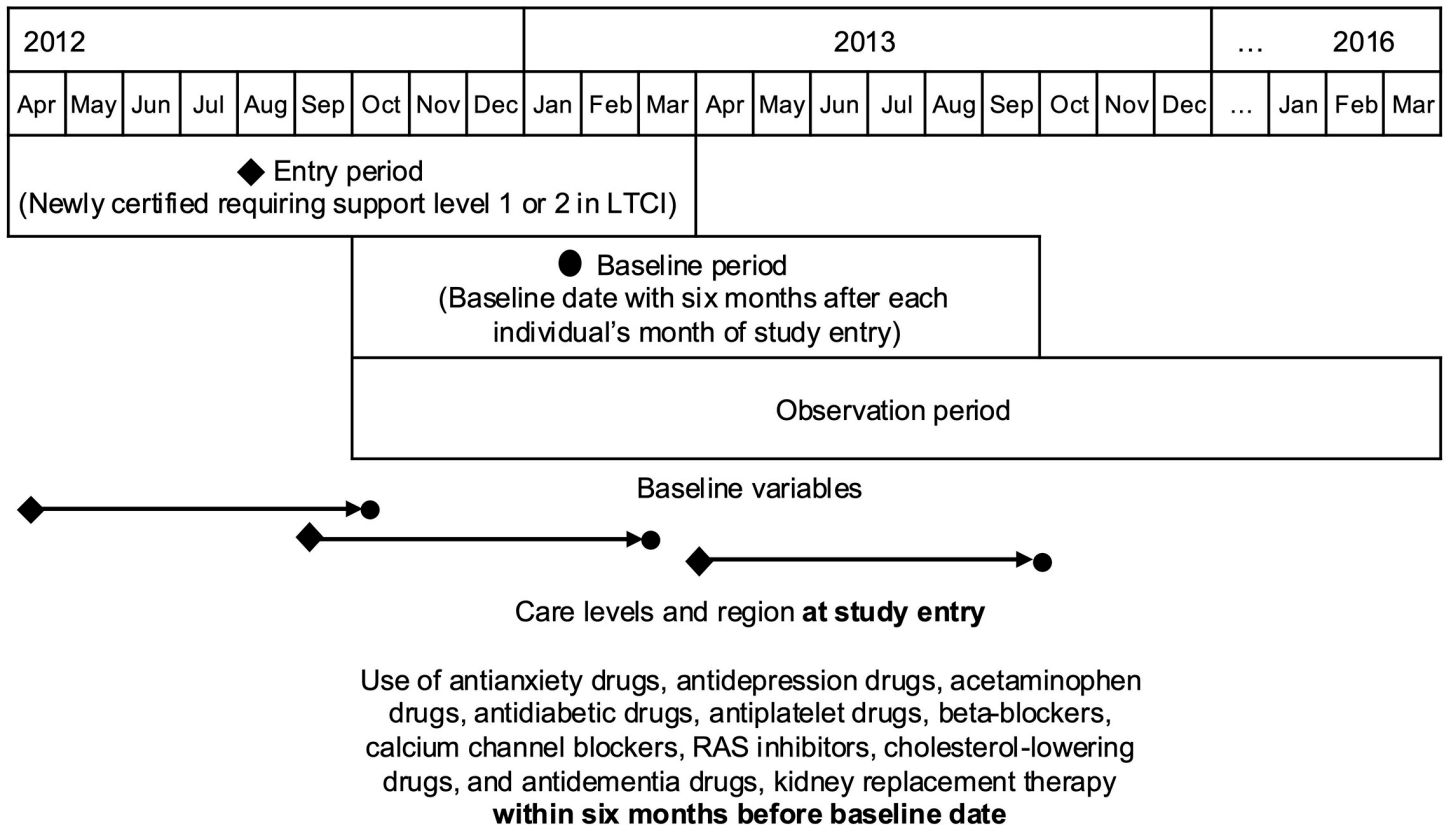
Measurements of the baseline and outcome variables.

The outcome of this study was the identification of functional disabilities obtained from LTCI certification in the KDB ^(27)^. Functional disabilities were identified when a person was newly qualified for LTCI level 1 or higher, based on a multistep assessment of functional and cognitive impairments by physicians and the Certification Committee of Needed Long-Term Care ^(21)^. The observational period was defined as the number of months from the baseline month to (i) LTCI certification, (ii) all-cause mortality, (iii) loss of insurance eligibility, or (iv) March 2016, whichever occurred first (Figure 2).

### Statistical analyses

We performed competing risk regression analyses using Fine and Gray proportional subdistribution hazards models, with death as a competing risk event. The cumulative probability of functional disability incidence was estimated using the cumulative incidence function estimator and compared using a weighted log-rank test ^(28)^. The association between sleeping medication use and the incidence of functional disability was evaluated using unadjusted and adjusted Fine and Gray proportional subdistribution hazards models ^(29)^. Subdistribution hazard ratios (SHRs) with 95% confidence intervals (CIs) for the sleeping medication use were calculated using unadjusted and multivariable-adjusted models, whereby covariates from each previous model were retained as follows: Model 1 was unadjusted; Model 2 included age, sex, and care level; Model 3 additionally included use of antianxiety drugs and antidepression drugs; Model 4 further included use of acetaminophen drugs, antidiabetic drugs, antiplatelet drugs, calcium channel blockers, renin-angiotensin system blockers, beta-blockers, cholesterol-lowering drugs, and antidementia drugs, kidney replacement therapy, and region.

To adjust for confounding due to participant characteristics, we used propensity scores (PS). To balance baseline characteristics between the sleeping medication use group and the no-use group, we conducted PS matching analysis. PS were estimated using a multivariable logistic regression model that included age, sex, baseline care level, kidney replacement therapy, and the use of antianxiety, antidepressant, acetaminophen, antidiabetic, antiplatelet, beta-blocker, calcium channel blocker, renin-angiotensin system blocker, cholesterol-lowering, and antidementia drugs as covariates. Each participant in the sleeping medication use group was matched to one participant in the no-use group using 1:1 nearest-neighbor greedy matching without replacement, with a caliper width of 0.2 standard deviations of the logit of the PS ^(30)^. Balance in baseline characteristics after matching was assessed using standardized mean differences (SMDs), with an SMD <0.1 indicating adequate balance ^(31)^. The incidence of functional disability was compared between the two groups using cumulative incidence function curves under competing risks and an unadjusted Fine-Gray subdistribution hazards model.

Hip fractures among older adults are known to result in functional disabilities ^(32)^. Furthermore, an association exists between sleeping medications and hip fractures ^(33)^. Hence, we conducted a sensitivity analysis to support the findings of the present study. In the sensitivity analyses, the association between sleeping medication use and the incidence of hip fracture was assessed using unadjusted and adjusted Fine and Gray proportional subdistribution hazards models.

Continuous variables were expressed as medians and interquartile ranges, and categorical variables were expressed as numbers and proportions. The level of significance was set at *p* < 0.05 unless otherwise specified. Statistical analyses were performed using R software, version 4.2.1.

## Results

The baseline characteristics of the 1,291 participants stratified by sleeping medication category, are presented in Table 1. Among 426 men, 293 (31.7%) did not use sleeping medication, and 133 (36.1%) used it at their baseline date. Among 865 women, 630 (68.3%) and 235 (63.9%) were classified into the “No Use” and “Use” groups, respectively. Participants using sleeping medication showed a higher prevalence of drug use and required kidney replacement therapy.

**Table 1.** 1,291 Participants’ Characteristics Stratified by Use of Sleeping Medications.

	Sleeping medications		p-Value
Baseline characteristics	No use	Use	
n	923	368	
Age, year	80 (74-84)	80 (76-84)	0.404
Men, n (%)	293 (31.7)	133 (36.1)	0.147
Baseline care levels, n (%)			
Requiring support 1	640 (69.3)	239 (64.9)	0.144
Requiring support 2	283 (30.7)	129 (35.1)	<0.001
Antianxiety drugs, n (%)	113 (12.2)	127 (34.5)	<0.001
Antidepression drugs, n (%)	25 (2.7)	34 (9.2)	<0.001
Acetaminophen, n (%)	23 (2.5)	18 (4.9)	<0.001
Antidiabetic drugs, n (%)	108 (11.7)	76 (20.7)	<0.001
Antiplatelet drugs, n (%)	229 (24.8)	163 (44.3)	<0.001
Beta-blockers, n (%)	67 (7.3)	63 (17.1)	<0.001
Calcium channel blockers, n (%)	305 (33.0)	198 (53.8)	<0.001
RAS blockers, n (%)	268 (29.0)	150 (40.8)	<0.001
Cholesterol-lowering drugs, n (%)	190 (20.6)	111 (30.2)	<0.001
Antidementia drugs, n (%)	68 (7.4)	31 (8.4)	0.597
Kidney replacement therapy, n (%)	8 (0.9)	10 (2.7)	0.022
Region, n (%)			
Izumi city	619 (67.1)	235 (63.9)	
Izumiotsu city	224 (24.3)	107 (29.1)	
Misaki town	80 (8.7)	26 (7.1)	

Data are presented as median (25%-75%) or n (%) RAS: renin-angiotensin system.

**Figure 3.**
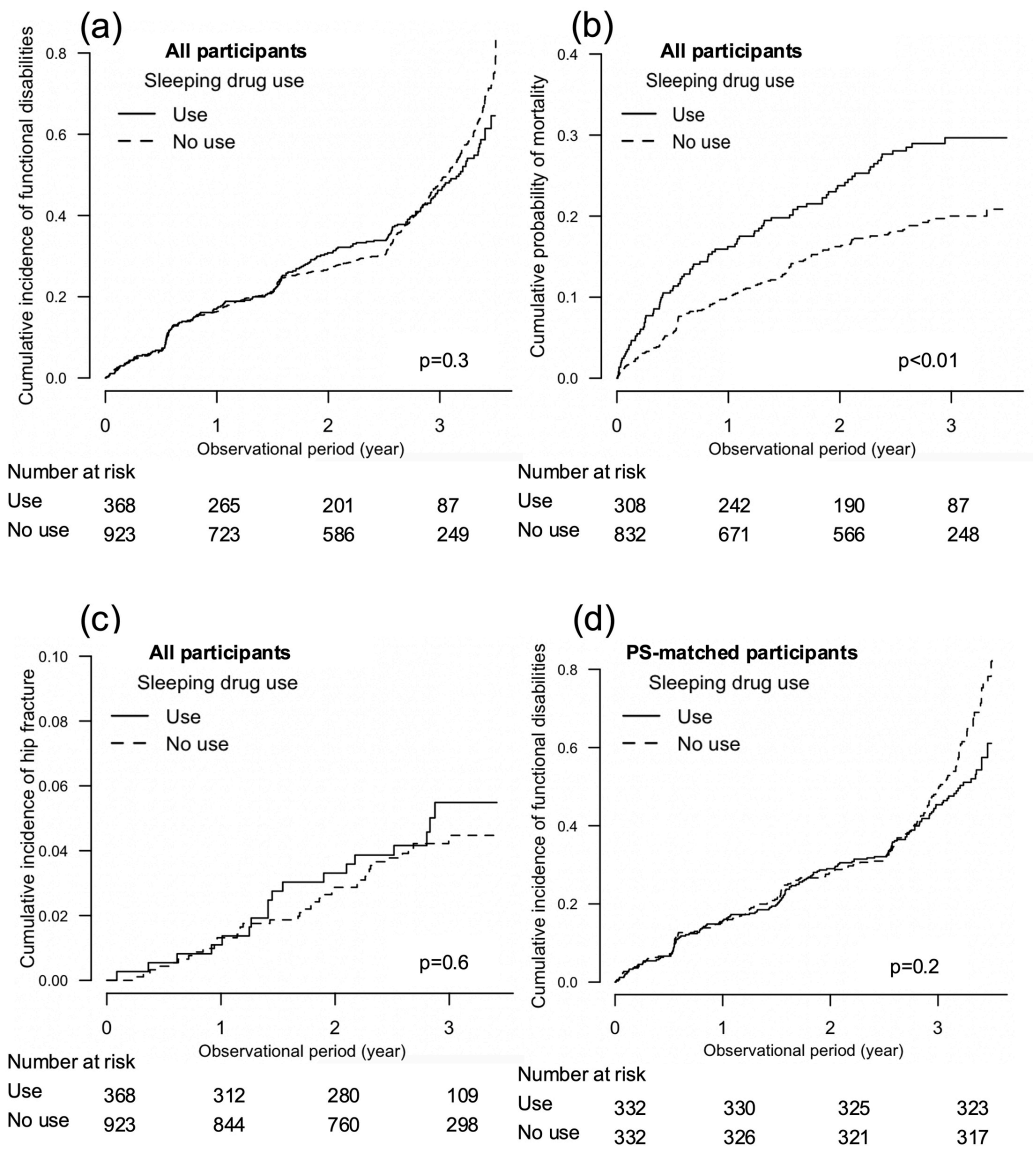
Cumulative incidence function curves for LTC needs under competing risks (a), Kaplan-Meier survival curves for all-cause mortality (b), and cumulative incidence function curves for hip fracture under competing risks (c) among 1,291 older adults aged ≥65 years with low care needs. Panel (d) shows cumulative incidence function curves for LTC needs under competing risks in 332 propensity score-matched pairs. LTC: long-term care.

During the median observational period of 2.5 years (interquartile range, 1.0-3.0), functional disability occurred in 504 (54.6%) and 184 (49.7%) participants in the “No Use” group and the “Use” group, respectively. The cumulative probability of functional disability was not significantly associated with sleeping medication use (Figure 3a). Although no clear difference was observed in the unadjusted cumulative incidence curves (Figure 3a), the association became apparent after adjustment for baseline characteristics and concomitant medication use in the multivariable Fine and Gray models. The unadjusted Fine and Gray model showed that the “No Use” and “Use” groups were not significantly associated with functional disability incidence (SHRs of “No Use” and “Use”: 1.00 [reference], 0.96 [CIs, 0.80-1.15] in Model 1) (Table 2). After adjusting for age, sex, and care level, “No Use” and “Use” groups were not significantly associated with the incidence of functional disabilities (SHRs of “No Use” and “Use”: 1.00 [reference], 0.91[CIs, 0.75-1.10] in Model 2). In addition, Model 3 was adjusted for the covariates included in Model 2, and for the use of antianxiety drugs, and antidepressant drugs. The “No use” and “Use” groups were not significantly associated with the incidence of functional disabilities (SHRs of “No Use” and “Use”: 1.00 [reference], 0.85[CIs, 0.70-1.03] in Model 3). Model 4 was adjusted for the covariates included in Model 3 and for the use of acetaminophen drugs, antidiabetic drugs, antiplatelet drugs, calcium channel blockers, renin-angiotensin system blockers, beta-blockers, cholesterol-lowering drugs, antidementia drugs, kidney replacement therapy, and region (SHRs of “No Use” and “Use”: 1.00 [reference], 0.82 [CIs, 0.68-0.99] in Model 4). The “Use” group in Model 4 was significantly associated with a lower incidence of functional disabilities than the “No Use” group after additional adjustments.

**Table 2.** Associations Between the Sleeping Medications and the Incidence of Functional Disabilities.

	All participants		PS-matched participants	
	Sleeping medications		Sleeping medications	
Variables	No use	Use	No use	Use
Number	923	368	332	332
Observational period, year	2.6 (1.2-3.0)	2.4 (0.8-3.0)	2.6 (1.4-3.0)	2.5 (0.9-3.0)
Incidence of functional disabilities, n (%)	504 (54.6)	183 (49.7)	197 (59.3)	157 (47.3)
All-cause mortality, n (%)	91 (9.9)	60 (16.3)	29 (8.7)	56 (16.9)
Model 1, SHR (95% CI)*	1.00 (reference)	0.96 (0.80-1.15)	1.00 (reference)	0.82 (0.66-1.03)
Model 2, SHR (95% CI)†	1.00 (reference)	0.91 (0.75-1.10)		
Model 3, SHR (95% CI)‡	1.00 (reference)	0.85 (0.70-1.03)		
Model 4, SHR (95% CI)§	1.00 (reference)	0.82 (0.68-0.99)		

CI: confidence interval; PS: propensity score SHR: sub-hazard ratio. *Model 1, unadjusted. †Model 2, adjusted for age (year), sex, care levels (requiring support 1; requiring support 2). ‡Model 3, adjusted for covariates in Model 2 and use of antianxiety drugs and antidepression drugs. §Model 4, adjusted for covariates in Model 3 and use of acetaminophen drugs, antidiabetic drugs, antiplatelet drugs, calcium channel blockers, renin-angiotensin system blockers, beta-blockers, cholesterol-lowering drugs, and antidementia drugs, kidney replacement therapy (hemodialysis, peritoneal dialysis, and kidney transplantation), and region (Izumi City, Izumiotsu City, and Misaki Town).

In the PS matching analysis, 332 matched pairs were generated. Baseline characteristics were well balanced between the two groups, with all SMDs <0.1 (Supplemental Table 1). Consistent with the findings from the unadjusted cumulative incidence function curves (Figure 3d) and the unadjusted Fine-Gray model (Table 2) in all participants, no significant association was observed between sleep medication use and the incidence of functional disability among PS-matched participants (SHRs for the “No Use” and “Use”: 1.00 [reference] and 0.82 [CIs, 0.66-1.03], respectively).

For the sensitivity analysis, the association between sleeping medication use and the incidence of hip fracture was assessed using unadjusted and adjusted Fine and Gray proportional subdistribution hazards models. The group using sleeping medications was not at significant increased risk of hip fracture (SHRs of “No Use” and “Use”: 1.00 [reference], 1.17 [CIs, 0.63-2.18] in Model 1; 1.00 [reference], 0.93 [CIs, 0.47-1.82] in Model 2; and 1.00 [reference], 0.92 [CIs, 0.47-1.79] in Model 3) (Supplemental Table 2).

## Discussion

This prospective cohort study, which included 1,291 older adults aged 65 years and older with low care needs in Japan’s LTCI during a three-year observational period, clarified that the group using sleeping medications had a significantly lower risk of functional disabilities than the group not using them. This finding differs from previous studies that showed an association between sleeping medications and functional disabilities and may provide useful suggestions for home care services for adults aged ≥65 years with low care needs.

Although previous studies have identified sleeping medication as a risk factor for functional disabilities, this study did not find an association between sleeping medication and functional disabilities among older adults. In Japan, the LTCI system actively offers preventive care for older adults with low care needs ^(11)^. Participants in this study received support from the LTCI system, and specialized care, such as safe living environment improvements provided by care providers, which may have prevented functional disabilities associated with sleeping medication use. In addition, the results of the present sensitivity analysis indicated no association between sleeping medication use and the incidence of hip fractures in these participants. When a hip fracture occurs in older adults, 25% become bedridden, 45% experience difficulty going out, and the reported mean Barthel Index is 49.6, indicating a high risk of functional disabilities ^(32)^. Professional fall prevention strategies focus on multiple risk factors, such as creating safe living environments and avoiding medications such as benzodiazepines that increase the risk of falls and hip fractures ^(34)^. For older adults with low care needs, receiving professional support may reduce the risk of potential functional disabilities.

The beneficial effects of sleeping medication use on functional disabilities in this study may be explained by circadian rhythms. Adjusting circadian rhythms increases daytime activity in older adults ^(35)^. Another cohort study that included 665 adults with low care needs reported that use of daycare services reduced the risk of functional disabilities by 40% ^(12)^. In contrast, a previous cohort study that included 256 adults in Japan revealed that sleep disorders were associated with a significantly high incidence of functional disabilities ^(36)^. Administration of sleeping medications can modulate circadian rhythms, thereby potentially increasing daytime activity levels and reducing the risk of functional disabilities. For individuals with low care needs, managing sleep with professional support, such as through assessment of sleeping medication dosage, may prevent functional disabilities.

In the fully adjusted model (Model 4), which included prescriptions for antidiabetic and cardiovascular medications, the association between sleeping medication use and the incidence of functional disability was attenuated. This observation suggests that differences in medical care, as reflected by the use of medications for diabetes and cardiovascular diseases, could partly account for the observed association. Previous studies have reported sleep disorders as risk factors for diabetes and cardiovascular disease ^(37)^, and more recent evidence has suggested an association with declines in physical function ^(38)^. Accordingly, individuals with diabetes or cardiovascular disease might be more likely to receive sleeping medications as part of routine clinical care. In this context, the lower incidence of functional disability observed among users of sleeping medications may reflect closer medical supervision or more comprehensive management of underlying conditions, rather than indicating a direct association between sleeping medication use and functional outcomes.

In this study, the Fine-Gray competing risk analysis showed a lower adjusted subdistribution hazard of functional disability among users of sleeping medications; however, all-cause mortality was higher in this group. Because death precludes the occurrence of functional disability, higher mortality may reduce the observed cumulative incidence of functional disability, even when death is explicitly accounted for as a competing event. Notably, the crude subdistribution hazard ratio was close to unity (0.96), whereas the association became apparent only after multivariable adjustment. This suggests that baseline differences in participant characteristics, care levels, and concomitant medication use may have masked the association in the unadjusted analysis. At the same time, the higher mortality in the sleeping medication-use group indicates that some individuals may have died before developing functional disability, which could partially explain the lower cumulative incidence observed in this group. Therefore, the lower adjusted subdistribution hazard should be interpreted cautiously and does not necessarily indicate a preventive role of sleeping medications against functional disability. Rather, the findings reflect the complex interplay between mortality, baseline health status, and functional outcomes in older adults with low care needs.

The present study has several limitations. First, this study was unable to conduct a detailed analysis of the type of sleeping medication because of the limited sample size of 368 individuals using sleeping medications. As the incidence of functional disabilities may differ by sleeping medication type ^(39), (40)^, future studies with larger sample sizes are necessary to assess the association between each sleeping medications and the incidence of functional disabilities. Second, the generalizability of the present findings should be examined in different cohorts because of differences in LTCI systems among countries. Several countries provide LTCI services to a more narrowly targeted population and offer these services less frequently compared with Japan ^(41)^. Third, the associations between sleeping medication use and the incidence of functional disabilities may be affected by unmeasured confounding factors, including daytime activity. A previous cross-sectional study using actigraphs reported that higher daytime activity was significantly associated with better functional performance ^(42)^. In addition, sleeping medications have been reported to increase daytime activity in older adults ^(15)^. Thus, the association between the use of sleeping medications and the incidence of functional disabilities in the present study may be confounded by differences in daytime activity levels. Moreover, although sleep disorders have been reported in previous studies as risk factors for diabetes and cardiovascular diseases ^(37), (43)^, we were unable to directly evaluate the presence of these comorbid conditions because of limitations inherent to the use of medical claims data. This limitation represents an important clinical consideration when interpreting the observed associations. Fourth, the observed association between sleeping medication use and the incidence of functional disability may be influenced by additional unmeasured confounding factors, including sleep disorders severity and baseline functional status. These factors are clinically important determinants of both medication use and functional outcomes but were not available in the medical claims database used in this study. As a result, confounding by indication cannot be excluded. Therefore, the lower adjusted incidence of functional disability observed among individuals using sleeping medications should be interpreted with caution. Well-designed prospective studies incorporating detailed assessments of sleep disorder severity and baseline functional status are needed to verify and clarify the findings of the present study. Fifth, sleeping medication use in this study was defined as having at least one prescription within a six-month period, without consideration of dosage, duration, or medication type. Because these factors are closely related to sleep disorder severity, this simplified exposure definition may have resulted in substantial misclassification. This represents a critical limitation of the present study and should be addressed in future research using more detailed exposure assessment.

In conclusion, this prospective cohort study of 1,291 adults aged 65 years with low care needs found that sleeping medication use was associated with a lower incidence of functional disability when death was considered a competing event. Although individuals using sleeping medications showed a lower adjusted subdistribution hazard of functional disability, all-cause mortality was higher in this group, indicating that the findings should be interpreted cautiously. These results highlight the importance of carefully managing sleep problems in adults with low care needs. Professional support, including appropriate assessment and management of sleeping medications, may be important for maintaining functional status in this population, rather than indicating a direct preventive role of sleeping medications.

## Article Information

### Authors Contributions

Conceptualization: Naoko Otsuki, Ryohei Yamamoto, and Ayumi Kono, Methodology: [Naoko Otsuki and Ryohei Yamamoto, Formal analysis: Naoko Otsuki, Investigation: Ayumi Kono, Writing - original draft preparation: Naoko Otsuki; Writing - review and editing: Ryohei Yamamoto and Ayumi Kono, Funding acquisition: Ayumi Kono, Resources: Ayumi Kono, Supervision: Ryohei Yamamoto and Ayumi Kono, Project administration: Ayumi Kono

### Conflicts of Interest

All authors have completed the ICMJE uniform disclosure form and declared: no support from any organization for submitted work; no financial relationships with any organizations that might have an interest in the submitted work in the previous three years; no other relationships or activities that could appear to have influenced the submitted work.

### Data Availability

The data presented in this study cannot be shared. These data originate from the municipal government of Izumi City, Izumiotsu City, and Misaki Town, and are not publicly available. Restrictions apply to the availability of these data, which are used under license and ethical approval. Inquiries regarding the data and analyses are to be directed to the corresponding author.

### Ethics Approval

This study was conducted in accordance with the ethical principles of the Declaration of Helsinki. This study was approved by the Institutional Review Boards of Osaka Metropolitan University Graduate School of Nursing (Approval Number 2023-04) and the Osaka University Health and Counseling Center (Approval Number 2023-07). The present study utilized anonymized data from the Kokuho database (KDB) provided by Izumi City, Izumiotsu City, and Misaki Town in Osaka Prefecture, Japan.

### Patient Consent

All data were anonymized by the local government, and the research team had no access to personally identifiable information. In accordance with the Ethical Guidelines for Medical and Biological Research Involving Human Subjects issued by the Ministry of Health, Labour and Welfare of Japan, informed consent was not required because this study analyzed only de-identified data from pre-existing medical claims records.

## Supplement

Supplementary Material
