# Supplementary material for: Sleeping Medications and Prevention of Functional Disability in Older Adults with Low Care Needs: A Prospective Cohort Study
Source: JMA J. 2026 Mar 27;9(3):660–8. doi: 10.31662/jmaj.2025-0534 (PMC13246268; doi:10.31662/jmaj.2025-0534)
Supplement: Supplementary Material [file 2433-3298-9-3-0660-s001.pdf]

Supplemental Table 1. Baseline characteristics of 332 pairs matched by propensity scores

	Sleeping medications		SMD
	No Use	Use	
Number	332	332	
Age, year	80 (76–85)	80 (76–85)	0.045
Men, n (%)	121 (36.4)	121 (36.4)	0.031
Baseline care levels, n (%)			0.013
Requiring support 1	207 (62.3)	217 (65.4)	
Requiring support 2	125 (37.7)	115 (34.6)	
Antianxiety drugs, n (%)	90 (27.1)	95 (28.6)	0.034
Antidepression drugs, n (%)	16 (4.8)	22 (6.6)	0.078
Acetaminophen, n (%)	17 (5.1)	14 (4.2)	0.043
Antidiabetic drugs, n (%)	70 (21.1)	62 (18.7)	0.060
Antiplatelet drugs, n (%)	142 (42.8)	139 (41.9)	0.018
Beta-blockers, n (%)	45 (13.6)	48 (14.5)	0.026
Calcium channel blockers, n (%)	175 (52.7)	170 (51.2)	0.030
RAS blockers, n (%)	144 (43.4)	131 (39.5)	0.080
Cholesterol-lowering drugs, n (%)	102 (30.7)	96 (28.9)	0.040
Antidementia drugs, n (%)	32 (9.6)	28 (8.4)	0.042
Kidney replacement therapy, n (%)	8 (2.4)	6 (1.8)	0.042

Data are presented as median (25%–75%) or n (%)

RAS, renin-angiotensin system

SMD, standardized mean differences

Supplemental Table 2. Associations between the use of sleeping medications and the incidence of hip fracture

	Sleeping medications	
	No use	Use
Number	923	368
Observational period, year	2.8 (2.5–3.1)	2.8 (2.1–3.1)
Incidence of hip fracture, n (%)	38 (4.1)	18 (4.9)
All-cause mortality, n (%)	149 (16.1)	89 (24.2)
Model 1, SHR (95% CI)*	1.00 (reference)	1.17 (0.63–2.18)
Model 2, SHR (95% CI)†	1.00 (reference)	0.93 (0.47–1.82)
Model 3, SHR (95% CI)‡	1.00 (reference)	0.92 (0.47–1.79)

SHR, sub-hazard ratio; CI, confidence interval

*Model 1, unadjusted; †Model 2, adjusted for age (year), sex, care levels (requiring support 1; requiring support 2), ‡Model 3, adjusted for covariates in Model 2 and use of antianxiety drugs and antidepression drugs
